# Bottom-Up Physiologically Based Oral Absorption Modeling of Free Weak Base Drugs

**DOI:** 10.3390/pharmaceutics12090844

**Published:** 2020-09-03

**Authors:** Naoya Matsumura, Asami Ono, Yoshiyuki Akiyama, Takuya Fujita, Kiyohiko Sugano

**Affiliations:** 1 Minase Research Institute, Ono Pharmaceutical Co., Ltd., 3-1-1 Sakurai, Shimamoto-cho, Mishima-gun, Osaka 618-8585, Japan; 2 Laboratory for Chemistry, Manufacturing, and Control, Pharmaceuticals Production & Technology Center, Asahi Kasei Pharma Corporation, 632-1 Mifuku, Izunokuni, Shizuoka 410-2321, Japan; ono.ar@om.asahi-kasei.co.jp; 3 Central Pharmaceutical Research Institute, Japan Tobacco Inc., 1-1 Murasaki-cho, Takatsuki, Osaka 569-1125, Japan; yoshiyuki.akiyama@jt.com; 4 Laboratory of Molecular Pharmacokinetics, College of Pharmaceutical Sciences, Ritsumeikan University, 1-1-1 Noji-higashi, Kusatsu, Shiga 525-8577, Japan; fujita-t@ph.ritsumei.ac.jp; 5 Molecular Pharmaceutics Lab., College of Pharmaceutical Sciences, Ritsumeikan University, 1-1-1 Noji-higashi, Kusatsu, Shiga 525-8577, Japan; suganok@fc.ritsumei.ac.jp

**Keywords:** free base, gastric dissolution, permeability, solubility, oral absorption, modeling

## Abstract

In this study, we systematically evaluated “bottom-up” physiologically based oral absorption modeling, focusing on free weak base drugs. The gastrointestinal unified theoretical framework (the GUT framework) was employed as a simple and transparent model. The oral absorption of poorly soluble free weak base drugs is affected by gastric pH. Alternation of bulk and solid surface pH by dissolving drug substances was considered in the model. Simple physicochemical properties such as pK_a_, the intrinsic solubility, and the bile micelle partition coefficient were used as input parameters. The fraction of a dose absorbed (Fa) in vivo was obtained by reanalyzing the pharmacokinetic data in the literature (15 drugs, a total of 85 Fa data). The AUC ratio with/without a gastric acid-reducing agent (AUCr) was collected from the literature (22 data). When gastric dissolution was neglected, Fa was underestimated (absolute average fold error (AAFE) = 1.85, average fold error (AFE) = 0.64). By considering gastric dissolution, predictability was improved (AAFE = 1.40, AFE = 1.04). AUCr was also appropriately predicted (AAFE = 1.54, AFE = 1.04). The Fa values of several drugs were slightly overestimated (less than 1.7-fold), probably due to neglecting particle growth in the small intestine. This modeling strategy will be of great importance for drug discovery and development.

## 1. Introduction

Recent drug candidates show poor solubility in aqueous media [1]. Because the oral absorption of a drug is affected by its solubility in the gastrointestinal (GI) tract, poorly soluble drugs often show incomplete, variable, and less than dose-proportional oral absorption. Therefore, it is important to accurately evaluate the oral absorption of a drug candidate at the preclinical stage and design a formulation suitable for clinical studies [2]. The oral absorption of poorly soluble drugs is susceptible to various physiological conditions in the GI tract, such as gastric pH, intestinal pH, buffer capacity, bile micelle concentration, gastrointestinal transit time, and hydrodynamics [3,4,5]. In the case of poorly soluble weak base drugs, gastric dissolution can have a marked impact on their oral absorption. Drug-drug interactions (DDI) with gastric acid-reducing agents (ARA), such as antacids, H_2_ blockers, and proton pump inhibitors, are of great clinical importance, as they could reduce the efficacy of a drug by interfering with gastric dissolution [6,7,8]. In addition, elderly subjects tend to be achlorhydric [9,10].

Physiologically based pharmacokinetic (PBPK) modeling has been anticipated to be a powerful tool to improve the productivity of drug discovery and development [11]. There have been many case studies of oral absorption PBPK modeling [12,13,14,15,16]. However, case studies are prone to publication bias. Therefore, systematic evaluation is required to avoid publication bias. In addition, case-by-case parameter fitting has been widely used in most of these publications. The simulation process should be standardized when evaluating the predictability of a model [17,18]. Publication bias and case-by-case parameter fitting reduce the reliability of the prediction model [19]. Recently, a commercial software product was systematically evaluated regarding ARA DDI prediction for weak base drugs [20]. The results suggest that scientific knowledge in this area is still premature (Figures 4 and 5 in [20]). In that study, free and salt forms were not differentiated as drug substances. However, free and salt forms exhibit completely different dissolution mechanisms.

In the stomach, free weak base drugs are dissolved by gastric acid. However, the dissolution rate becomes relatively slow because the acidity is neutralized by the dissolving free bases at the solid surface (discussed in detail in the theory section) [21,22,23,24]. Some portion of the particles remain undissolved in the stomach and reach the small intestine. These particles serve as seed particles for particle growth, which reduces the drug concentration in the small intestine [25,26]. On the other hand, in the case of salt forms, the dissolution rate is predominantly determined by the solubility product (K_sp_) [27]. In addition, the nucleation of free base particles occurs before starting precipitation in the small intestine [28]. However, there are many unknown factors in the nucleation process [29,30,31,32,33].

In this study, we systematically evaluated an oral absorption PBPK model for poorly soluble weak base drugs, focusing on the free forms for the first time. To the best of our knowledge, there has been no systematically validated model for weak base drugs. The gastrointestinal unified theoretical framework (the GUT framework) was employed as a simple and transparent model. Previously, the GUT framework has been systematically validated for neutral and acidic drugs by the consortium of biopharmaceutical tools (CoBiTo) in Japan [34,35]. The model equations and physiological parameters of the GUT framework have been fully disclosed and well documented in the literature [5,34,35,36]. In this study, a simple approximate analytical solution that describes the gastric dissolution processes of free weak base drugs was introduced to the GUT framework. The fraction of a dose absorbed (Fa) and the ARA effect were predicted from simple input parameters such as pK_a_, the octanol-water partition coefficient (logP_oct_), and the biorelevant solubility, in a fully “bottom-up” manner.

## 2. Theory

### 2.1. Solubility in the Stomach

Gastric fluid is acidified by hydrochloric acid (HCl). Because HCl is not an efficient buffer, the pH value increases when a free weak base drug is added to the gastric fluid [37]. The equilibrium pH value after the addition of an excess amount of a free weak base drug (pH_eq_) can be calculated as follows. From charge neutrality at equilibrium,

(1) $$\left[H^{+}\right]+\left[{\mathrm{BH}}^{+}\right]=\left[{\mathrm{Cl}}^{-}\right]+\left[{\mathrm{OH}}^{-}\right]$$

where B is a free base drug. From the definition of the dissociation constant (K_a_) and the water ion product constant (K_w_),

(2) $$K_{a}=\frac{\left[H^{+}\right]{\left[B\right]}_{\mathrm{sat}}}{\left[{\mathrm{BH}}^{+}\right]}=\frac{\left[H^{+}\right]S_{0}}{\left[{\mathrm{BH}}^{+}\right]}$$

(3) $$K_{w}=\left[H^{+}\right]\left[{\mathrm{OH}}^{-}\right]$$

where S_0_ is the intrinsic solubility of a free base drug (= [B]_sat_). The S_0_ value can be back-calculated from the buffer solubility and pK_a_. From charge neutrality in an initial HCl solution,

(4) $$\left[{\mathrm{Cl}}^{-}\right]+{\left[{\mathrm{OH}}^{-}\right]}_{\mathrm{ini}}={\left[H^{+}\right]}_{\mathrm{ini}}$$

where [H^+^]_ini_ is the initial proton concentration that can be calculated from the initial gastric pH (pH_STini_). By inserting Equations (2) to (4) into Equation (1),

(5) $$\left[H^{+}\right]+\frac{\left[H^{+}\right]S_{0}}{K_{a}}={\left[H^{+}\right]}_{\mathrm{ini}}-\frac{K_{w}}{{\left[H^{+}\right]}_{\mathrm{ini}}}+\frac{K_{w}}{\left[H^{+}\right]}$$

this equation can be rearranged to a quadratic equation of [H^+^].

(6) $${\left[H^{+}\right]}^{2}\left(1+\frac{S_{0}}{K_{a}}\right)-\left[H^{+}\right]\left({\left[H^{+}\right]}_{\mathrm{ini}}-\frac{K_{w}}{{\left[H^{+}\right]}_{\mathrm{ini}}}\right)-K_{w}=0$$

By solving this equation, the equilibrium pH value (pH_eq_) can be calculated. From pH_eq_ and the Henderson–Hasselbalch equation, the maximum solubility of a free weak base drug in the stomach (S_ST_) can be calculated as,

(7) $$S_{\mathrm{ST}}=S_{0}\left(1+\frac{{\left[H^{+}\right]}_{\mathrm{eq}}}{K_{a}}\right)$$

where [H^+^]_eq_ is the proton concentration of the solution in equilibrium with a free weak base drug.

The dose number in the stomach (Do_ST_) is defined as,

(8) $${\mathrm{Do}}_{\mathrm{ST}}=\frac{\mathrm{Dose}}{S_{\mathrm{ST}}V_{\mathrm{ST}}}$$

where Dose is the dose amount and V_ST_ is the gastric fluid volume.

### 2.2. Dissolution Rate in the Stomach

In the case of free weak base drugs, solid surface pH (pH_surface_) becomes higher than bulk pH. The pH_surface_ value can be calculated using the Mooney–Stella equation [21,22,23,24]. The solid surface solubility (S_surface, ST_) can be calculated from pH_surface_ and S_0_, similar to Equation (7). The dissolution rate coefficient in the stomach (k_diss, ST_) can be calculated as,

(9) $$k_{\mathrm{diss},\mathrm{ST}}=\frac{3{\mathrm{DS}}_{\mathrm{surface},\mathrm{ST}}}{\rho }\overset{i}{\sum }\frac{f_{i}}{r_{p,i}^{2}}\;$$

where D_ST_ is the diffusion coefficient in the gastric fluid, ρ is the true density of a drug substance (set to 1.2 g/cm^3^), f_i_ is the fraction of a drug amount in a particle size bin (i), and r_p,i_ is the initial particle radius. The particle size distribution was assumed to have a log-normal distribution with ln2 standard deviation. The diffusion coefficient of a monomer drug molecule is calculated as,

(10) $$D\left({\mathrm{cm}}^{2}/s\right)=9.9\times 10^{-5}{\mathrm{MW}}^{-0.453}$$

The dissolution number in the stomach (Dn_ST_) can be calculated as,

(11) $${\mathrm{Dn}}_{\mathrm{ST}}=k_{\mathrm{diss},\mathrm{ST}}\cdot T_{\mathrm{ST}}$$

where T_ST_ is the gastric transit time.

### 2.3. Fraction Dissolved in the Stomach

The fraction dissolved in the stomach (Fd_ST_) for the solubility-limited (SL) and the dissolution rate-limited (DRL) cases can be calculated as,

(12) $${\mathrm{Fd}}_{\mathrm{ST}}\left(\mathrm{for}\;\mathrm{SL}\right)=\frac{1}{{\mathrm{Do}}_{\mathrm{ST}}}$$

(13) $${\mathrm{Fd}}_{\mathrm{ST}}\left(\mathrm{for}\;\mathrm{DRL}\right)=\frac{k_{\mathrm{diss},\mathrm{ST}}}{k_{\mathrm{diss},\mathrm{ST}}+k_{\mathrm{tr},\mathrm{ST}}}=\frac{1}{1+\frac{k_{\mathrm{tr},\mathrm{ST}}}{k_{\mathrm{diss},\mathrm{ST}}}}=\frac{1}{1+\frac{1}{{\mathrm{Dn}}_{\mathrm{ST}}}}$$

where k_tr, ST_ is the gastric transit rate constant (= 1/T_ST_). From Equations (12) and (13), an approximate solution for Fd_ST_ for general cases can be derived as,

(14) $${\mathrm{Fd}}_{\mathrm{ST}}=\frac{1}{{\mathrm{Do}}_{\mathrm{ST}}+1+\frac{1}{{\mathrm{Dn}}_{\mathrm{ST}}}}$$

### 2.4. Calculation of the Fraction of a Dose Absorbed Considering Gastric Dissolution

This study hypothesized that drug molecules dissolved in the stomach would not rapidly precipitate in the small intestine before absorption (the validity of this assumption is discussed later). In addition, drug absorption from the stomach was assumed to be negligible. In this case, the fraction of a dose absorbed (Fa) attributed to gastric dissolution (Fa_FdST_) becomes

(15) $${\mathrm{Fa}}_{\mathrm{FdST}}={\mathrm{Fd}}_{\mathrm{ST}}\left(1-\exp\left(-{\mathrm{Pn}}_{\mathrm{SI}}\right)\right)$$

where Pn_SI_ is the permeation number in the small intestine. The total Fa is the sum of Fa_FdST_ and the fraction of a dose absorbed in the small intestine attributed to drug dissolution in the small intestine (Fa_SI_).

(16) $$\mathrm{Fa}={\mathrm{Fa}}_{\mathrm{FdST}}+\left(1\;-{\;\mathrm{Fd}}_{\mathrm{ST}}\right){\mathrm{Fa}}_{\mathrm{SI}}$$

Fa_SI_ can be calculated by the Fa equation as [4,38],

(17) $${\mathrm{Fa}}_{\mathrm{SI}}=1-\exp\left(-\frac{1}{\frac{1}{{\mathrm{Dn}}_{\mathrm{SI}}}+\frac{{\mathrm{Do}}_{\mathrm{SI}}}{{\mathrm{Pn}}_{\mathrm{SI}}}}{\mathrm{Tn}}_{\mathrm{SI}}\right){\;\mathrm{if}\;\mathrm{Do}}_{\mathrm{SI}}<1,{\;\mathrm{set}\;\mathrm{Do}}_{\mathrm{SI}}=1$$

where Do_SI_ is the dose number, Dn_SI_ is the dissolution number, and Tn_SI_ is the transit time number in the small intestine. The Do_SI_ value is calculated from the undissolved drug amount reaching the small intestine (Dose (1 − Fd_ST_)). The details of Fa_SI_ calculation have been well documented in the literature and are summarized in Supplementary Material 1. Even though dynamic simulations can also be performed by the GUT framework [26], to improve the transparency of the calculation processes, the Fa equation is used in this study. The contribution of gastric dissolution on Fa (Fa_FdST_%) is calculated as Fa_FdST_/Fa × 100.

To evaluate the effect of gastric dissolution, Fa was predicted by the following three scenarios regarding gastric dissolution:

(A) No gastric dissolution
(B) Using bulk pH
(C) Using pH_surface_ and pH_eq_.

In the case of (A), Fd_ST_ was set to 0 (so that Fa = Fa_SI_). In the case of (B), the initial gastric pH (pH_ini_) in Table 1 was used instead of pH_eq_ in the S_ST_ calculation (Equation (7)). In addition, S_surface,ST_ was set equal to S_ST._ In the case of (C), pH_eq_ and S_ST_ were calculated from S_0_, pK_a_, and pH_ini_ (Equations (1) to (7)). pH_surface_ was calculated by the Mooney–Stella equation from S_0_, pK_a_, the diffusion coefficient of a drug (D, Equation (10)), and pH_ini_. S_surface,ST_ was then calculated from S_0_, pK_a_, and pH_surface_. This was a fully bottom-up prediction; therefore, no parameter fitting was performed based on in vivo data.

The details of the calculation scheme and all intermediate values, including pH_eq_, pH_surface_, S_ST_, S_surface,ST_, and the effective intestinal membrane permeability (P_eff_), are provided in Supplementary Material 1.

### 2.5. Fa Rate-Limiting Steps (FaRLS)

The oral absorption of a drug can be categorized as permeability, dissolution rate, and solubility-permeability limited cases (PL, DRL, and SL, respectively). Permeability can be further divided into epithelial membrane permeability-limited and unstirred water layer permeability-limited (-E and -U, respectively). The criteria of FaRLS are summarized in Supplementary Material 1 Table S1. Similarly, the limiting factor of Fd_ST_ was categorized as DRL or SL based on Equation (14).

### 2.6. Physiological Parameters

The physiological parameters are summarized in Table 1. The gastric emptying half-life (T_ST1/2_) values were set to 10 and 60 min for fasted and fed state humans [39,40,41,42,43,44], and 10 and 180 min for fasted and fed dogs [45,46,47,48], respectively. The time-averaged gastric fluid volume (V_ST_) was set to 125 and 250 mL for fasted and fed state humans, respectively, considering water and food intake with a drug and the gastric emptying of the stomach contents [39,40,41]. Similarly, the gastric fluid volume in fasted dogs was set to 50 mL, considering water intake with a drug [49]. The gastric fluid volume in fed dogs has not been found in the literature. Therefore, the gastric fluid volume in fed dogs was assumed to be 250 mL as the size of the stomach is comparable to humans [50]. Dogs consume about 200–400 g of food once a day [51,52].

The gastric pH values were set to 2.0 and 2.7 for the fasted and fed state humans, respectively. It is well known that the gastric pH value temporarily increases to about pH 6 after food intake. However, the pH value returns to acidic in 60–90 min due to postprandial gastric acid secretion [10,53,54]. Therefore, the gastric pH value at the mean gastric transit time was used for the fed state in humans (pH 2.7). The gastric pH value in the fasted state ranges from pH 1.0 to 2.4 in humans, with a mean value of about pH 1.5 [10,54]. The pH value would be slightly increased by water intake. Therefore, the gastric pH value in the fasted state was set to pH 2.0. The gastric pH value in dogs was set to pH 2.0 for both the fasted and fed states, respectively [55,56]. However, it should be noted that the gastric pH value in dogs shows a large variation [57,58,59]. The gastric pH value after the administration of PPI or H_2_ blockers was set to pH 6.0 for both humans and dogs [60,61]. The small intestinal pH values in fasted and fed dogs were set to be 7.0 and 6.0, respectively [34,62]. The other physiological parameters of the small intestine were the same as used in the previous studies [34,36].

### 2.7. In Vivo Fa Data

In vivo Fa values were calculated from the literature data. As there is no exact method to measure Fa, one or a few approximations have been used to estimate Fa from in vivo PK data [36,63].

(I) Fa described in the literature was used as it was.
(II) Relative bioavailability of solution vs. solid form formulation.
(III) Relative bioavailability in the fasted state vs. in the fed state (especially when Do_SI_ < 1 in the fed state).
(IV) Relative bioavailability with the low vs. high pH stomach when Do_SI_ < 1 in the stomach (for basic drugs) (not used in this study)
(V) Dose-normalized relative bioavailability at Do_SI_ < 1 vs. Do_SI_ > 1 when the terminal elimination half-life is consistent.
(VI) From absolute bioavailability (F) and hepatic clearance using Fa = F/(1 − CLh/Qh).

The methods (II)–(V) have been used because Fa can be assumed to be 100% when there are no solubility and dissolution rate limitations for lipophilic drugs (logD_oct_ at pH 6.5 > 0.5) [5]. In the cases when multiple calculation methods could have been applied, these methods showed similar Fa values (within 0.8- to 1.3-fold difference in this study), suggesting that these methods are appropriate in most cases. The AUC ratio with/without ARA (AUCr) was also calculated from the literature data. Full references for in vivo Fa and AUCr are provided in Supplementary Material 2 Table S2. In addition to human Fa data, dog Fa data were also collected from the literature. Dogs have been used as a preclinical animal model for formulation studies [51,64,65].

### 2.8. Statistics

The Fa predictability was evaluated by the absolute average fold error (AAFE), the average fold error (AFE), the coefficient of determination (r^2^), and the percentage within twofold error. The absolute average fold error (AAFE) was calculated as,

(18) $$\mathrm{AAFE}=10^{\left(\frac{\sum^{\text{​}}\left|\log\left(\frac{\mathrm{Predicted}\;\mathrm{Fa}}{\mathrm{Observed}\;\mathrm{Fa}}\right)\right|}{n}\right)}$$

The AAFE converts negative log fold errors to positive values before averaging, measuring the spread of the predictions. The average fold error (AFE) was calculated as,

(19) $$\mathrm{AFE}=10^{\left(\frac{\sum \log\left(\frac{\mathrm{Predicted}\;\mathrm{Fa}}{\mathrm{Observed}\;\mathrm{Fa}}\right)}{n}\right)}$$

AFE indicates whether there is a trend for underprediction (AFE < 1) or overprediction (AFE > 1).

## 3. Results

### 3.1. Model Drugs

An extensive literature survey was performed to collect in vivo Fa data of poorly soluble weak base drugs (free form). The Fa values of 15 drugs were calculated from the literature data (a total of 85 Fa data). The majority of weak base drugs are launched as a salt form [27]. However, as mentioned in the introduction, the Fa prediction of salt form drugs is out of the scope of this study. The physicochemical properties of model drugs are shown in Table 2. The pK_a_ and logP_oct_ values ranged from 3.8 to 7.5 and −0.2 to 5.7, respectively, covering the practical range of poorly soluble weak base drugs.

### 3.2. Prediction Model

Appropriate Fa_SI_ prediction is the prerequisite for predicting the Fa values of free weak base drugs. As mentioned in the introduction, prior to the present study, the GUT framework has been validated for predicting Fa_SI_ in humans and dogs [34,35]. In these studies, neutral and free weak acid drugs were mainly used because the Fa values of these drugs are little affected by gastric dissolution (Fa ≈ Fa_SI_). Structurally diverse drugs were employed in those studies (for example, 27 drugs with a total of 96 in vivo Fa values were employed in [35]). The GUT framework demonstrated the sufficient Fa_SI_ predictability necessary for the present study.

### 3.3. Results of Fa Prediction

**Figure 1:**
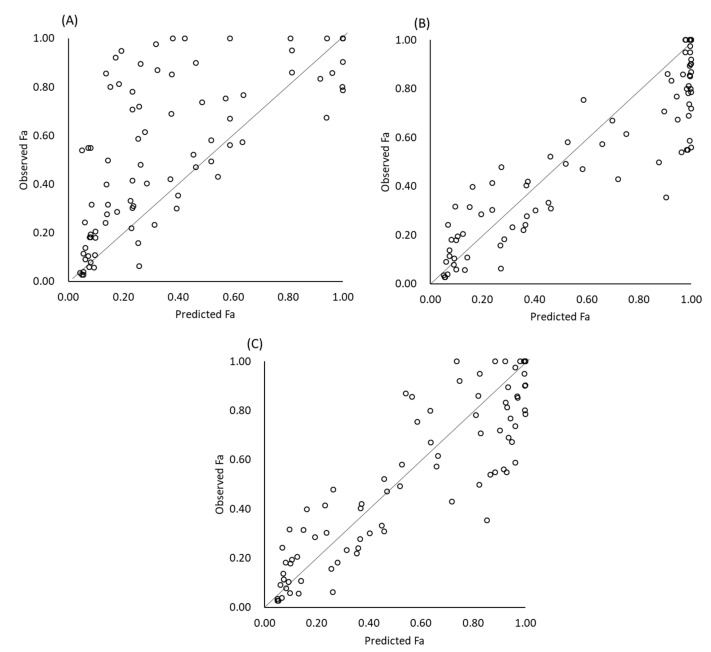
Predicted and observed Fa. (**A**) No gastric dissolution, (**B**) using bulk pH, and (**C**) using pH_surface_ and pH_eq_.

**Figure 2:**
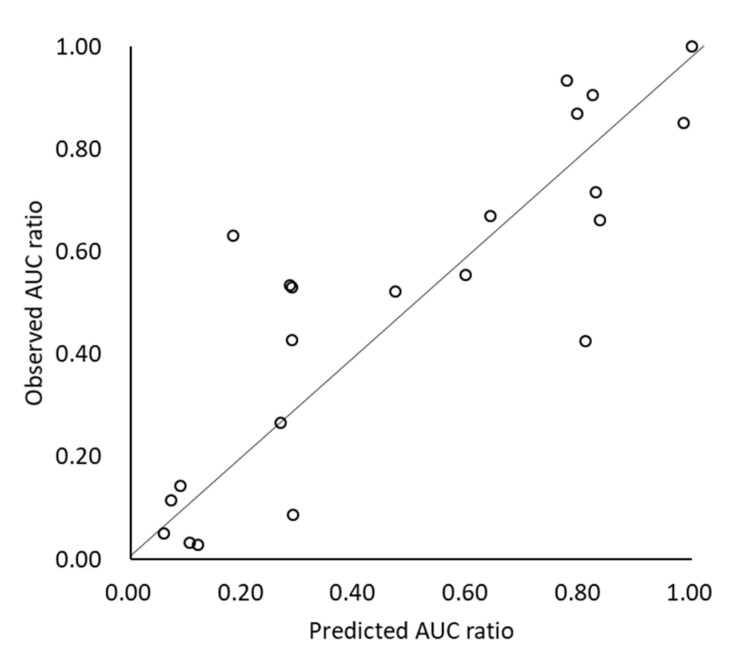
Predicted and observed AUCr with/without ARA.

The results of Fa prediction are shown in Table 3 and Figure 1 and Figure 2. When gastric dissolution was neglected, the in vivo Fa values of free weak base drugs were markedly underestimated (Figure 1) (Table 4). By taking gastric dissolution into account, predictability was markedly improved. When the initial bulk pH was used to calculate gastric dissolution, the Fa values were overestimated. This overestimation was reduced by using pH_eq_ and pH_surface_. The Fa values of posaconazole were exceptionally underestimated. When posaconazole was excluded, a slight overestimation was observed, even when pH_eq_ and pH_surface_ were taken into account (AFE = 1.15).

### 3.4. Prediction of Drug-Drug Interaction (DDI) with Acid-Reducing Agents (ARA)

The effect of the ARA (AUCr) was also appropriately predicted by scenario (C) (AAFE = 1.54, AFE = 1.04, r^2^ = 0.73, % within 2-fold error = 82%) (Figure 2). When gastric dissolution was limited by the dissolution rate (DRL), the drug tended to show marked AUC reduction by ARA DDI (>30% reduction in all DRL cases). In the case when gastric dissolution was solubility-limited (SL), the contribution of gastric dissolution on Fa became small, and therefore, ARA DDI became less significant.

## 4. Discussion

In this study, a simple model equation describing the gastric dissolution processes was introduced for the first time. By using this modeling strategy, both Fa and AUCr of free weak base drugs were successfully predicted in a bottom-up manner from the intrinsic drug parameters and physiological parameters. The Fa predictability observed in this study (AAFE = 1.40, AFE = 1.04) was similar to the previously reported values for neutral and free acid drugs (AAFE = 1.46, AFE = 0.95) [35]. This good predictability suggests that the simple Fd_ST_ and Fa models appropriately capture the essence of oral drug absorption. The results of this study suggest that, instead of using the bulk pH, pH_eq_ and pH_surface_ should be used so as not to overestimate gastric dissolution. In many cases, gastric pH was predicted to be increased from the initial value of pH 2.0 to above pH 3.0 by dissolving free base molecules (Supplementary Material 3). In addition, the surface pH was also predicted to be increased to above pH 3.0 in many cases. A difference of 1 unit of pH corresponds to a 10-fold difference in solubility and dissolution rates. Therefore, it is important to consider pH_eq_ and pH_surface_ when simulating the gastric dissolution of free weak base drugs. 

However, even when using pH_eq_ and pH_surface_, the Fa values of aprepitant (100 mg, 5 μm), cinnarizine, danixirin, and dipyridamole were slightly overestimated (1.6-, 1.4-, 1.6-, and 1.7-fold, respectively), probably because particle growth and precipitation in the small intestine were neglected. This overestimation is in good agreement with the previous modeling study [26], in which particle growth in the small intestine was considered.

In the case of free weak base drugs, some portion of drug particles would remain undissolved in the stomach and reach the small intestine. These particles can serve as seed particles for particle growth, which reduces dissolved drug concentration in the small intestine [25,26]. However, Koyama et al. reported that the effect of seed particles can be less significant in some drugs [29]. In addition, the rate of concentration reduction became slower as the size of the seed particles became larger [29]. In the case of high permeability drugs, the intestinal permeation rate can be faster than the particle growth rate [92]. Most of the model drugs of this study are lipophilic, so that they can rapidly permeate the intestinal membrane. Therefore, for lipophilic drugs, particle growth in the small intestine may only have a slight effect on Fa.

The drug concentration in the small intestine can also be reduced by precipitation via nucleation without seed particles. However, direct clinical observation suggested that precipitation in the small intestine was minimal (dipyridamole, ≤7%) or limited (ketoconazole, ≤16%) after solution administration [93]. In addition, rapid intestinal absorption reduces supersaturation and prevents rapid precipitation in the small intestine [94,95,96]. Furthermore, in vivo intestinal fluid components such as mucus can inhibit drug precipitation [97]. Taken together, in the case of free weak base drugs, it can be a good approximation to neglect particle growth and precipitation. However, intestinal precipitation could be significant for salt form drugs because it can induce higher supersaturation than free form drugs [98]. Another possible reason for overestimation is a deviation from the theoretical pH–solubility profile. The predicted solubility value of cinnarizine at pH_surface_ (11 mg/mL at pH 2.6) is about 7-fold higher than the experimental value (1.5 mg/mL at pH 2.5) [99]. The pH–solubility profile of cinnarizine suggested that this is not due to the common ionic effect [99].

In this study, the Fa values of high dose posaconazole were two to threefold underestimated. The reason is not clear. Previously, Hens et al. reported that after intragastric administration of posaconazole (40 mg, 1/10 of clinical dose) as acidified suspension (pH 1.6, 240 mL, 76% in solution), supersaturation was observed for 45 min. However, when administered as an unbuffered suspension in tap water (pH 7.1, 240 mL), no supersaturation was observed [100]. In the present study, at the clinical doses of 200 and 400 mg, less than 10% was predicted to be dissolved in the stomach. Therefore, the small intestinal process may be the reason for the underestimation.

The simple “bottom-up” modeling strategy employed in this study is suitable for use in drug discovery, where available input data are limited. Because particle growth in the small intestine is neglected, the predicted Fa value should be interpreted as a possible maximum Fa value. In addition, the predicted AUCr value should also be considered as a worst-case scenario. This predictive property is rather preferable to avoid false-negative DDI predictions. Another advantage of this simple modeling strategy is that it can provide insight into the rate-limiting step of gastric dissolution. The gastric dissolution can be diagnosed as SL and DRL by Equation (14). In addition, the contribution of gastric dissolution on Fa can be estimated by Equation (16). This information is important for formulation design. In addition, because simple analytical solutions enable tracing calculations and improve transparency, this modeling strategy is also suitable for regulatory submission.

For more accurate prediction, a complicated numerical simulation might be required to describe particle dissolution in the stomach, particle transit from the stomach to the small intestine, and particle growth and redissolution in the small intestine. Previously, it was suggested that the Fa values of dipyridamole could be more appropriately predicted when particle growth in the small intestine was considered [26]. Systematic validation of the GUT framework with dynamic simulation is under investigation and will be published elsewhere. Commercial software products such as GastroPlus^™^ and SimCYP^®^ can also handle gastric dissolution processes [20]. The predictability of these software products should be systematically evaluated before they are used for regulatory purposes [35]. In this study, nearly 100 Fa and AUCr values were compiled from the literature. These datasets would be an asset for the evaluation of simulation models in the future. The predictability of the simple analytical solution in this study will serve as a benchmark for assessing the prediction skills of more complex prediction models [35]. It should be noted that the good descriptive power of a complex model does not necessarily imply its good predictive skills. The prediction skill of a model should be carefully investigated before using the model in drug discovery and development.

## 5. Conclusions

In this study, the bottom-up modeling strategy to predict the Fa values of free weak base drugs was systematically evaluated. By considering gastric dissolution, the in vivo Fa values were appropriately predicted (AAFE = 1.40, AFE = 1.04). In addition, the AUC ratios with/without ARA were also appropriately predicted (AAFE = 1.54, AFE = 1.04). The Fa predictability confirmed in this study is comparable with that previously reported for neutral and free acid drugs (AAFE = 1.46, AFE = 0.95) [35]. The modeling strategy employed in this study will be of great importance for drug discovery and development.

## Figures and Tables

**Table 1:** Physiological parameters.

Parameters	Human		Dog	
	Fasted	Fed	Fasted	Fed
Stomach ^1^				
Initial gastric pH, pH_STini_ (normal/ with ARA)	2.0/6.0	2.7/6.0	2.0/6.0	2.0/6.0
Gastric emptying half-life, T_ST1/2_ (min) ^2^	10	60	10	180
Gastric fluid volume, V_ST_ (mL)	125	250	50	250
Small intestine ^3^				
Small intestinal pH	6.5	6.0	7.0	6.0
Small intestinal bile micelle concentration, C_bm_ (mM)	3.0	15	5.0	18
Small intestinal transit time, T_SI_ (min)	210	210	120	120
Small intestinal fluid volume, V_SI_ (mL)	130	156	19	22
Plicate expansion, PE	3	3	1	1
Villi expansion, VE	10	10	10	10
Radius of small intestine, R_GI_ (cm)	1.5	1.5	0.5	0.5
Degree of flatness, DF	1.7	1.7	1.7	1.7
Paracellular radius, R_ParaMW_	8.46	8.46	12.9	12.9
Unstirred water layer thickness, h_UWL_ (cm)	0.03	0.03	0.03	0.03
Particle-drifting coefficient, C_pd_	2.2	2.2	2.2	2.2
Nominal pore radius, R_mucus_ (μm)	2.9	2.9	2.9	2.9

^1^ References are cited in Supplementary Material 1; ^2^ Gastric transit time (T_ST_) = T_ST1/2_/ ln2; ^3^ The same values used in the previous studies [34,36]. A detailed explanation of these parameters is provided in Supplementary Material 1.

**Table 2:** Physicochemical properties of free weak base drugs.

Drugs	MW	pK_a_	logP_oct_	Solubility (mg/mL)			Ref.
				Buffer ^3^	FaSSIF ^4^	FeSSIF ^4^	
Acalabrutinib	466	5.8	2.0	0.048 (pH 8.0)	0.12 ^5^		[66]
Albendazole	265	4.2	3.1	0.0009	0.0021		[67,68]
Aprepitant	534	4.2	4.8	0.0008	0.019, 0.021 ^7^	0.119	[69,70,71]
BMS	550	6.3	2.3	0.019 (pH 6.6)	0.043		[72]
Cinnarizine	369	7.5	5.7	0.0014	0.013	0.112	[73]
Danixirin	442	4.8	1.2 ^1^	0.004 (pH 6.0)	0.009		[74]
Dasatinib	488	6.8	3.2	0.01 (pH 6.0)	0.032		[75,76]
Dipyridamole	505	4.9	3.9	0.006	0.017		[63,77]
Enoxacin	320	6.2	−0.2 ^2^	0.38	0.54 ^5^	0.83 (pH 5.8) ^5^	[20,78,79]
Etoricoxib	359	4.6	3.1	0.16	0.16		[80]
Gefitinib	447	7.2	4.1	0.002 (pH 6.9)	0.085 (pH 6.4)	2.0 (pH 5.4)	[81]
Iburtinib	441	3.8	4.0	0.0021 (pH 6.8)	0.016 (pH 7.0)		[82,83]
Ketoconazole	531	6.5	4.3	0.012	0.021		[63]
Palbociclib	448	7.4	1.3 ^2^	0.009 (pH 7.9) ^6^			[84,85,86]
Posaconazole	701	4.6	5.4	0.0001	0.005		[87,88]

^1^ Calculated by ACD/logP (Advanced Chemistry Development, Inc.); ^2^ Enoxacin: Caco-2 6.4 × 10^−6^ cm/s at pH 6.5 [89]; Palbociclib: MDCK 45 × 10^−6^ cm/s at pH 7.4 [84]. These values were used for intrinsic permeability calculation; ^3^ pH 6.5 unless otherwise noted; ^4^ Fasted state simulated intestinal fluid (FaSSIF: taurocholic acid 3 mM, egg lecithin 0.75 mM, pH 6.5). Fed state simulated intestinal fluid (FeSSIF: taurocholic acid 15 mM, egg lecithin 3.75 mM, pH 5.0). The solubility values in these fluids were used unless otherwise noted; ^5^ FaSSIF v2: taurocholic acid 3 mM, egg lecithin 0.2 mM, pH 6.5, FeSSIF v2: taurocholic acid 10 mM, egg lecithin 2 mM, pH 5.8 [90]; ^6^ The solubility in the small intestine was estimated from S_0_ and logP_oct_ (see Supplementary Material 1); ^7^ Dog FaSSIF: taurocholic acid 5 mM, egg lecithin 1.25 mM, pH 6.5 [71].

**Table 3:** Dose, particle size, species, and food state for simulation and in vivo Fa data.

Drugs	Physiological Condition	Dose (mg)	d (μm)	Predicted					Observed	
				Fa			AUCr	Fa_FdST_%	Fa	AUCr
				(A) ^1^	(B) ^1^	(C) ^1^	(C) ^1^	(C) ^1,2^		
Acalabrutinib	Human, Fasted	100	98	0.54	1.00	0.94		88% (DRL)		
	Human, Fasted, ARA	100	98	0.54	0.57	0.56	0.60	6% (SL)		0.56
Albendazole	Human, Fasted	350	3.4	0.10	0.14	0.14		32% (SL)	0.11	
	Human, Fasted	400	3.4	0.09	0.13	0.13		30% (SL)	0.06	
	Human, Fed	400	3.4	0.18	0.19	0.19		8% (SL)	0.29	
	Human, Fasted	700	3.4	0.08	0.10	0.10		23% (SL)	0.06	
	Human, Fasted	800	3.4	0.07	0.09	0.09		22% (SL)	0.10	
	Human, Fasted	1400	3.4	0.05	0.07	0.07		17% (SL)	0.04	
	Human, Fasted, ARA	1400	3.4	0.05	0.05	0.05	0.83	0% (SL)	0.03	0.72
	Human, Fasted	2100	3.4	0.04	0.05	0.05		15% (SL)	0.03	
Aprepitant	Human, Fasted	40	0.12	1.00	1.00	1.00		28% (SL)	0.90	
	Human, Fasted	100	5.0	0.23	0.36	0.35		38% (SL)	0.22	
	Human, Fasted	125	0.12	1.00	1.00	1.00		11% (SL)	0.79	
	Human, Fasted	250	0.12	0.96	0.97	0.97		6% (SL)	0.86	
	Human, Fasted	375	0.12	0.94	0.95	0.95		4% (SL)	0.67	
	Human, Fasted	500	0.12	0.92	0.92	0.92		3% (SL)	0.83	
	Human, Fed	125	0.12	1.00	1.00	1.00		5% (SL)	1.00	
	Dog, Fasted	20	0.12	0.94	0.98	0.98		24% (SL)	1.00	
	Dog, Fed	20	0.12	1.00	1.00	1.00		61% (DRL)	1.00	
	Dog, Fasted	20	0.48	0.54	0.72	0.72		33% (SL)	0.43	
	Dog, Fasted	20	1.9	0.24	0.46	0.46		52% (SL)	0.31	
	Dog, Fasted	20	2.0	0.23	0.45	0.45		53% (SL)	0.33	
	Dog, Fasted	20	5.0	0.14	0.37	0.37		63% (SL)	0.28	
	Dog, Fasted	20	5.5	0.14	0.36	0.36		64% (SL)	0.24	
	Dog, Fed	20	5.5	0.64	0.95	0.94		64% (DRL)	0.77	
	Dog, Fasted	20	25	0.08	0.28	0.28		74% (SL)	0.18	
BMS	Human, Fasted	150	3.0	0.80	1.00	1.00		82% (DRL)		
	Human, Fasted, ARA	150	3.0	0.80	0.82	0.81	0.81	1% (SL)		0.43
	Dog, Fasted	150	40	0.08	1.00	0.70		91% (DRL)		
	Dog, Fasted, ARA	150	40	0.08	0.09	0.08	0.12	5% (SL)		0.03
Cinnarizine	Human, Fasted	25	25	0.25	0.99	0.96		98% (DRL)	0.59	
	Human, Fasted, ARA	25	25	0.25	0.27	0.26	0.27	1% (SL)	0.16	0.27
	Human, Fasted	25	60	0.08	0.99	0.93		99% (DRL)	0.55	
	Human, Fasted, ARA	25	60	0.08	0.09	0.08	0.09	2% (SL)	0.08	0.14
	Human, Fasted	50	25	0.18	0.99	0.93		96% (DRL)	0.81	
	Human, Fed	50	25	0.38	1.00	0.92		83% (DRL)	1.00	
	Human, Fasted	50	60	0.07	0.98	0.89		99% (DRL)	0.55	
	Human, Fed	50	60	0.19	0.98	0.82		92% (DRL)	0.95	
	Dog, Fasted	25	25	0.05	0.96	0.87		97% (DRL)	0.54	
	Dog, Fasted, ARA	25	25	0.05	0.06	0.05	0.06	2% (SL)	0.03	0.05
Danixirin	Human, Fed	50	2.0	0.40	0.91	0.85		70% (DRL)	0.35	
	Human, Fasted	100	1.8	0.23	0.90	0.83		80% (DRL)	0.71	
	Human, Fasted, ARA	100	1.8	0.23	0.24	0.24	0.29	2% (SL)	0.30	0.43
	Human, Fed	100	1.8	0.28	0.75	0.66		65% (SL)	0.61	
Dasatinib	Human, Fasted	50	27	0.38	1.00	0.97		94% (DRL)	0.85	
	Human, Fasted	70	27	0.32	1.00	0.96		92% (DRL)	0.98	
	Human, Fasted	100	27	0.26	0.99	0.93		91% (DRL)	0.90	
	Human, Fasted, ARA	100	27	0.26	0.27	0.26	0.28	1% (SL)	0.48	0.54
Dipyridamole	Human, Fasted	50	75	0.14	0.88	0.82		95% (DRL)	0.50	
	Human, Fasted, ARA	50	75	0.14	0.15	0.15	0.18	5% (DRL)	0.31	0.63
	Dog, Fasted	50	75	0.04	0.78	0.68		96% (DRL)		
	Dog, Fasted, ARA	50	75	0.04	0.05	0.05	0.07	9% (SL)		0.11
Enoxacin	Human, Fasted	400	25	0.59	0.98	0.89		57% (DRL)	1.00	
	Human, Fed	400	25	0.81	1.00	0.99		36% (SL)	1.00	
	Human, Fasted, ARA	400	25	0.59	0.70	0.64	0.64	11% (SL)	0.67	0.67
Etoricoxib	Human, Fasted	120	40	1.00	1.00	1.00		78% (DRL)	1.00	
	Human, Fasted, ARA	120	40	1.00	1.00	1.00	1.00	13% (SL)	1.00	1
Gefitinib	Human, Fasted	50	30	0.49	0.99	0.96		94% (DRL)	0.74	
	Human, Fasted	100	30	0.38	0.99	0.94		89% (DRL)	0.69	
	Human, Fasted	250	30	0.23	0.99	0.81		83% (DRL)	0.78	
	Human, Fasted, ARA	250	30	0.23	0.24	0.23	0.29	0% (SL)	0.41	0.53
	Human, Fed	250	30	1.00	1.00	1.00		46% (SL)	0.80	
	Human, Fasted	500	30	0.15	0.98	0.64		81% (DRL)	0.80	
Ibrutinib	Human, Fasted	560	10	0.10	0.13	0.13		21% (SL)	0.21	
	Human, Fasted, ARA	560	10	0.10	0.10	0.10	0.80	0% (SL)	0.18	0.87
	Human, Fed	560	10	0.39	0.40	0.40		3% (SL)	0.30	
Ketoconazole	Human, Fasted	200	25	0.26	1.00	0.90		84% (DRL)	0.72	
	Human, Fasted, ARA	200	25	0.26	0.27	0.26	0.29	2% (SL)	0.06 ^3^	0.09 ^3^
	Human, Fed	200	25	0.59	1.00	0.92		61% (DRL)	0.56	
	Human, Fasted	400	25	0.17	1.00	0.75		83% (DRL)	0.92	
	Human, Fed	400	25	0.42	1.00	0.74		53% (SL)	1.00	
	Human, Fasted	800	25	0.14	0.99	0.57		79% (SL)	0.86	
	Human, Fed	800	25	0.32	1.00	0.54		45% (SL)	0.87	
	Dog, Fasted	200	25	0.06	0.99	0.62		91% (DRL)		
	Dog, Fasted, ARA	200	25	0.06	0.07	0.06	0.10	3% (SL)		0.03
Palbociclib	Human, Fasted	125	16	0.46	1.00	1.00		82% (DRL)	0.90	
	Human, Fasted, ARA	125	16	0.46	0.58	0.47	0.47	2% (SL)	0.47	0.52
	Human, Fed	125	16	0.81	1.00	1.00		64% (DRL)	0.95	
	Human, Fed, ARA	125	16	0.81	0.91	0.82	0.82	2% (SL)	0.86	0.91
Posaconazole	Human, Fed	50	1.7	0.63	0.66	0.66		6% (SL)	0.57	
	Human, Fed	100	1.7	0.57	0.59	0.59		3% (SL)	0.75	
	Human, Fasted	200	1.7	0.08	0.11	0.11		23% (SL)	0.19	
	Human, Fasted, ARA	200	1.7	0.08	0.08	0.08	0.78	0% (SL)	0.18	0.93
	Human, Fed	200	1.7	0.52	0.53	0.53		2% (SL)	0.58	
	Human, Fed, ARA	200	1.7	0.52	0.52	0.52	0.99	0% (SL)	0.49	0.85
	Human, Fasted	400	1.7	0.06	0.07	0.07		17% (SL)	0.14	
	Human, Fasted, ARA	400	1.7	0.06	0.06	0.06	0.84	0% (SL)	0.09	0.66
	Human, Fed	400	1.7	0.46	0.46	0.46		1% (SL)	0.52	
	Human, Fed	800	1.7	0.37	0.37	0.37		1% (SL)	0.42	
	Human, Fed	1200	1.7	0.31	0.32	0.32		1% (SL)	0.23	
	Dog, Fasted	100	1.7	0.05	0.07	0.07		26% (SL)	0.11	
	Dog, Fed	100	1.7	0.29	0.37	0.37		24% (SL)	0.40	
	Dog, Fed	400	1.7	0.14	0.16	0.16		15% (SL)	0.40	
	Dog, Fed	800	1.7	0.08	0.10	0.10		13% (SL)	0.32	
	Dog, Fed	1200	1.7	0.06	0.07	0.07		12% (SL)	0.24	

^1^ (A) No gastric dissolution, (B) using bulk pH, and (C) using pH_surface_ and pH_eq_; ^2^ The parentheses indicate the limiting factor of gastric dissolution; ^3^ This in vivo Fa value could be an underestimated value due to steep nonlinearity in intestinal metabolism [91].

**Table 4:** Statistics of Fa prediction.

Statistics	Prediction Scenario ^1^		
	(A) No Gastric Dissolution	(B) Bulk pH	(C) pH_eq_/pH_surface_
All drug			
N	85	85	85
AAFE	1.85	1.41	1.40
AFE	0.64	1.10	1.04
r^2^	0.47	0.83	0.79
% within 2-fold error	64	89	91
Without posaconazole			
N	69	69	69
AAFE	1.87	1.37	1.36
AFE	0.64	1.23	1.15
r^2^	0.43	0.83	0.78
% within 2-fold error	65	93	94

^1^ (A) No gastric dissolution, (B) using bulk pH, and (C) using pH_surface_ and pH_eq_.

## Supplementary Materials

The following are available online at https://www.mdpi.com/1999-4923/12/9/844/s1, Supplementary Material 1: The GUT framework for Fa_SI_ calculation, Supplementary Material 2: In vivo Fa values and references, Supplementary Material 3: Intermediate calculation parameters.
