# Supplementary material for: Bottom-Up Physiologically Based Oral Absorption Modeling of Free Weak Base Drugs
Source: Pharmaceutics. 2020 Sep 3;12(9):844. doi: 10.3390/pharmaceutics12090844 (PMC7558956; doi:10.3390/pharmaceutics12090844)
Supplement: Supplementary file 1 [file pharmaceutics-12-00844-s001.zip › Supplementary Materials/Supplementary Materials 1_ The GUT framework for FaSI calculation_v2.docx]

Supplementary Materials: Bottom-Up Physiologically Based Oral Absorption Modeling of Free Weak Base Drugs

Naoya Matsumura, Asami Ono, Yoshiyuki Akiyama, Takuya Fujita and Kiyohiko Sugano

**ABBREVIATIONS**

AAFE, absolute average fold error

AFE, average fold error

ARA, acid-reducing agents

AUCr, AUC ratio with/without ARA

C_bm_, small intestinal bile micelle concentration

CoBiTo, consortium of biopharmaceutics tools

C_pd_, particle-drifting coefficient

C_water_, concentration of water

D_bm_, diffusion coefficient of bile micelle bound molecules

DDI, drug-drug interaction

DF, degree of flatness

D_mono_, diffusion coefficient of monomer molecules

Dn_SI_, dissolution number in the small intestine

Dn_ST_, dissolution number in the stomach

Do_SI_, dose number in the small intestine

Do_ST_, dose number in the stomach

DRL, dissolution rate limited case

D_SI_, diffusion coefficient in the small intestinal fluid

D_ST_, diffusion coefficient in the gastric fluid

f_0_, fraction of undissociated species

Fa, fraction of a dose absorbed

Fa_FdST_, Fa attributed to gastric dissolution

FaRLS, Fa rate-limiting step

Fa_SI_, Fa in the small intestine

FaSSIF, fasted state simulated intestinal fluid

Fd_ST_, fraction dissolved in the stomach

FeSSIF, fed state simulated intestinal fluid

f_mono_, fraction of unbound species

F_oral_, oral absolute bioavailability

GI, gastrointestinal

GUT, gastrointestinal unified theoretical

h_eff_, effective thickness of the UWL

h_fam_, thickness of the firmly adhered mucus

HH, Henderson-Hasselbalch

h_pd_, thickness of particle drifting region

h_UWL_, unstirred water layer thickness

K_a_, definition of the dissociation constant

K_bm_, bile micelle-water partition coefficient

k_diss, SI_, dissolution rate coefficient in the small intestine

k_diss, ST_, dissolution rate coefficient in the stomach

k_perm_, permeation rate coefficient

K_sp_, solubility product

k_t_, colon exited fraction

k_tr, ST_, gastric transit rate constant (= 1/T_ST_)

K_w_, water ion-product constant

logP_oct_, octanol-water partition coefficient

MW, molecular weight

P_app_, apparent permeation coefficient

PBPK, physiologically-based pharmacokinetic

PDE, particle drifting effect

PE, plicate expansion

P_eff_, effective intestinal membrane permeability

P_ep_’, effective epithelial membrane permeability

pH_eq_, equilibrium pH

pH_STini_, initial gastric pH

pH_surface_, solid surface pH

PL, permeability limited case

Pn, permeation number in the small intestine

P_trans0_, intrinsic passive transcellular permeability

P_UWL_, UWL permeability

P_WC_, water conveyance

r^2^, coefficient of determination

R_GI_, radius of small intestine

R_mucus_, nominal pore radius

r_p, I_, initial particle radius

R_ParaMW_, nominal radius of the paracellular pathway

R_SA_, surface area ratio of the membrane and the particles in the UWL

S_dissolv_, dissolved drug concentration in the small intestinal fluid

S_0_, intrinsic solubility

SL, solubility-permeability limited case

S_ST_, maximum solubility in the stomach

S_surface, ST_, solid surface solubility

Tn_SI_, transit time number in the small intestine

T_SI_, small intestinal transit time

T_ST_, gastric transit time

T_ST1/2_, gastric emptying half-life

UWL, unstirred water layer

VE, villi expansion

V_SI_, small intestinal fluid volume

V_ST_, gastric fluid volum

**Fa_SI_ calculation**

Fa_SI_ can be calculated from Do_SI_, Dn_SI_, and Pn_SI_ by Equation (1) (Equation 17 in the main document) [1].

| $\mathrm{Fa}_{\mathrm{SI}}=1-exp\left( -\frac{1}{\frac{1}{\mathrm{Dn}_{\mathrm{SI}}}+\frac{\mathrm{Do}_{\mathrm{SI}}}{\mathrm{Pn}_{\mathrm{SI}}}}\mathrm{Tn}_{\mathrm{SI}} \right)\mathrm{if}\mathrm{Do}_{\mathrm{SI}}<1, set \mathrm{Do}_{\mathrm{SI}}=1$ | (1) |
| --- | --- |
| $\mathrm{Do}_{\mathrm{SI}}=\frac{\mathrm{Dose}}{S_{\mathrm{dissolv}}{\cdot V}_{\mathrm{SI}}}$ | (2) |
| $\mathrm{Dn}_{\mathrm{SI}}=k_{diss, ST}\cdot T_{\mathrm{SI}}$ | (3) |
| $\mathrm{Pn}=k_{\mathrm{perm}}\cdot T_{\mathrm{SI}}$ | (4) |

where Do_SI_ is the dose number, Dn_SI_ is the dissolution number, Pn is the permeation number and Tn_SI_ is the transit time number in the small intestine [2], Dose is the dose strength, S_dissolv_ is the dissolved drug concentration in the small intestinal fluid, V_SI_ is the small intestinal fluid volume, k_diss, ST_ is the dissolution rate coefficient, T_SI_ is the small intestinal transit time, and k_perm_ is the permeation rate coefficient.

The S_dissolv_ value in the small intestine was calculated using the physiological pH and bile micelle concentration (C_bm_) in the small intestine. The intrinsic solubility (S_0_) was back-calculated from the measured solubility in blank FaSSIF (S_blank_) and pK_a_ by the Henderson-Hasselbalch (HH) equation. The bile micelle-water partition coefficient for undissociated (K_bm,0_) and charged species (K_bm,+_ and K_bm,-_ for monocation and monoanion, respectively) were also back-calculated by the modified HH equation (Eqs. 5 and 6) [3].

| $S_{\mathrm{dissolv}}=S_{0}\left( 1+\frac{\left[ H^{+} \right]}{\mathrm{Ka}}+\frac{C_{\mathrm{bile}}}{C_{\mathrm{water}}}・K_{bm,0}+\frac{\left[ H^{+} \right]}{\mathrm{Ka}}・\frac{C_{\mathrm{bile}}}{C_{\mathrm{water}}}・K_{bm,+} \right) for a base$ | (5) |
| --- | --- |
| $S_{\mathrm{dissolv}}=S_{0}\left( 1+\frac{\mathrm{Ka}}{\left[ H^{+} \right]}+\frac{C_{\mathrm{bile}}}{C_{\mathrm{water}}}・K_{bm,0}+\frac{\mathrm{Ka}}{\left[ H^{+} \right]}・\frac{C_{\mathrm{bile}}}{C_{\mathrm{water}}}・K_{bm,-} \right) for an acid$ | (6) |

where C_water_ is the concentration of water (55.6 mol/L). The K_bm,+_ and K_bm,-_ were estimated as,

| logK_bm,+_ = logK_bm_ − 1 | (7) |
| --- | --- |
| logK_bm,−_ = log_Kbm_ − 2 | (8) |

The K_bm_ values in the fasted state and the fed state were calculated using the solubility values in FaSSIF and FeSSIF, respectively. When the solubility in these media is not available, K_bm_ can be estimated from logP_oct_ as logK_bm_ = 0.74 logP_oct_ + 2.29 [4].

The k_diss, SI_ value was calculated by the Nernst-Brunner-Noyes-Whitney equation assuming spherical particles.

| $k_{diss, SI}=\frac{3D_{\mathrm{SI}}\cdot S_{\mathrm{dissolv}}}{\rho}\sum^{i} \frac{f_{i}}{r_{p,i}^{2}}$ | (9) |
| --- | --- |

where D_SI_ is the effective diffusion coefficient in the small intestinal fluid, ρ is the true density of a drug substance (set to 1.2 g/cm^3^), f_i_ is the fraction of a drug amount in a particle size bin (i), and r_p,i_ is the initial particle radius. The particle size distribution was assumed to have a log-normal distribution with ln2 standard deviation. The D_SI_ value was calculated as,

| $D_{\mathrm{SI}}=D_{\mathrm{mono}}\cdot f_{\mathrm{mono}}+D_{\mathrm{bm}}\left( 1-f_{\mathrm{mono}} \right)$ | (10) |
| --- | --- |
| $f_{\mathrm{mono}}=\frac{S_{\mathrm{blank}}}{S_{\mathrm{dissolv}}}$ | (11) |

where D_mono_ is the diffusion coefficient of monomer molecules, and D_bm_ is the diffusion coefficient of bile micelle bound molecules calculated from the bile acid concentration as previously reported [5]. The D_bm_ value in the unstirred water layer (UWL) adjacent to the intestinal membrane was set to be 3-fold larger than that in the bulk media. The D_mono_ value was calculated from the molecular weight as [6, 7],

| $D_{\mathrm{mono}}\left( \mathrm{cm}^{2}/s \right)=9.9\times{10}^{-5}\mathrm{MW}^{-0.453}$ | (12) |
| --- | --- |

The k_perm_ value was calculated as follows [8].

| $k_{\mathrm{perm}}=\frac{2DF}{R_{\mathrm{GI}}}\cdot P_{\mathrm{eff}}$ | (13) |
| --- | --- |
| $P_{\mathrm{eff}}=\frac{\mathrm{PE}}{\frac{1}{{P^{'}}_{\mathrm{ep}}}+\frac{1}{P_{\mathrm{UWL}}}}=\frac{\mathrm{PE}}{\frac{1}{{VE\cdot f}_{\mathrm{mono}}\left( f_{0}P_{trans0}+P_{\mathrm{para}} \right)}+\frac{1}{\frac{D_{\mathrm{eff}}}{h_{\mathrm{eff}}}+P_{\mathrm{WC}}}}$ | (14) |

where DF is the degree of flatness, R_GI_ is the radius of the small intestine, P_eff_ is the effective intestinal membrane permeability, PE is the plica expansion factor, P_ep_’ is the effective epithelial membrane permeability, P_UWL_ is the UWL permeability, VE is the villi expansion factor, f_mono_ is the fraction of unbound species, f_0_ is the fraction of undissociated species, P_trans0_ is the intrinsic passive transcellular permeability, P_para_ is the paracellular permeability, h_eff_ is the effective thickness of the UWL, and P_WC_ is the water conveyance. The f_0_ value was calculated by the HH equation. The P_trans0_ and P_para_ values were calculated as follows [8-10].

| $P_{trans0}\left( \mathrm{cm}/s \right)=2.36\times{10}^{-6}{P_{\mathrm{oct}}}^{1.1}$ | (15) |
| --- | --- |
| $P_{\mathrm{para}}\left( \mathrm{cm}/s \right)=3.9\times{10}^{-4}・\frac{1}{\mathrm{MW}^{1/3}}・\mathrm{RK}\left( \frac{\mathrm{MW}^{1/3}}{R_{\mathrm{ParaMW}}} \right)\times\left( f_{0} + \sum^{Z(Z\neq0)} f_{z}・\frac{2.39・Z}{1-e^{-2.39・z}} \right)$ | (16) |
| $\mathrm{RK}\left( x \right)=\left( 1-x \right)^{2}\left[ 1-2.104\left( x \right)+2.09\left( x \right)^{3}-0.95\left( x \right)^{5} \right] x<1$ | (17) |

where R_ParaMW_ is the nominal radius of the paracellular pathway defined based on MW^1/3^ (8.6 for humans and 12.9 for dogs) [11], RK is the Renkin function which represents the sieving effect of pores, and f_z_ is the fraction of the z-charged molecular species (z = −1, 0, +1).

The h_eff_ value was calculated considering that particles drifted into the UWL would reduce the effective thickness of UWL (the particle drifting effect (PDE)) [12].

| $h_{\mathrm{eff}}=h_{\mathrm{fam}}\cdot\left( 1-RK\left( \frac{r_{p,mean}}{R_{\mathrm{mucus}}} \right) \right)+h_{\mathrm{pd}}-\frac{1}{2}h_{\mathrm{pd}}\cdot R_{\mathrm{SA}} R_{\mathrm{SA}}\leq1$ | (18) |
| --- | --- |
| $h_{\mathrm{eff}}=h_{\mathrm{fam}}\cdot\left( 1-RK\left( \frac{r_{p,mean}}{R_{\mathrm{mucus}}} \right) \right)+\frac{1}{2}\cdot\frac{h_{\mathrm{pd}}}{R_{\mathrm{SA}}} R_{\mathrm{SA}}>1$ | (19) |
| $R_{\mathrm{SA}}=\frac{3{\cdot C}_{\mathrm{pd}}\cdot h_{\mathrm{pd}}\cdot Dose}{V_{\mathrm{GI}}\cdot\rho}\sum_{i} \frac{f_{i}}{r_{p,i}}$ | (20) |

where h_fam_ is the thickness of the firmly adhered mucus, h_pd_ is the thickness of particle drifting region defined as h_pd_ = h_UWL_ - h_fam_, R_mucus_ is the nominal pore radius of mucus layer, R_SA_ is the surface area ratio of the membrane and the particles in the UWL, and C_pd_ is the particle drifting coefficient. When neglecting PDE, the h_eff_ value was set equal to the h_UWL_ value.

A dimensionless number which express the prolonged duration of saturated concentration (Tn_SI_) was introduced as [1],

| $\mathrm{Tn}_{\mathrm{SI}}=1- \frac{1}{{k_{t}・T}_{\mathrm{SI}}}\ln\left( \frac{1}{1 + \frac{\mathrm{Do}_{\mathrm{SI}}\mathrm{Dn}_{\mathrm{SI}}}{\mathrm{Pn}}} \right) \mathrm{Do}_{\mathrm{SI}} ・\left( 1-EXT\left( \mathrm{Tn}_{\mathrm{SI}} \right) \right) > 1$ | (21) |
| --- | --- |
| $\mathrm{Tn}_{\mathrm{SI}} =1- \frac{1}{{k_{t}・T}_{\mathrm{SI}}}\ln\left( \frac{1}{1 - \frac{1}{\mathrm{Do}_{\mathrm{SI}}}}-1 \right)\mathrm{if}\mathrm{Do}_{\mathrm{SI}}<1, set \mathrm{Tn}_{\mathrm{SI}}=1$ | (22) |

where the k_t_ value for the colon exited fraction expressed by the sigmoidal curve is 1.31 h^-1^ for humans and 2.6 h^-1^ for dogs. Do(1-EXT(Tn_SI_)) is the Do at time t considering the colon exit of the dosed drug particles. The smaller value of Equation (21) or (22) should be taken as Tn_SI_. The minimum value of Tn_SI_ is 1.

The default physiological parameters provided in the GUT framework were used for Fa prediction (Table 1 in the main document).

**Fa Rate-Limiting Step (FaRLS)**

The oral absorption of a drug can be categorized as permeability, dissolution rate and solubility-permeability limited cases (PL, DRL and SL, respectively). Permeability can be further divided to epithelial membrane permeability limited and UWL permeability limited (-E and -U, respectively). The criteria for each class of FaRLS are summarized in Table S1 [13, 14].

**Table S1.** Fa Rate Limiting Step (FaRLS)^a.^

| **Rate limiting step** | **Abbreviation** | **Conditions** |  |  |
| --- | --- | --- | --- | --- |
| Dissolution rate limited | DRL | Dn < Pn/Do ^b^ |  |  |
| Epithelial membrane permeability limited | PL-E | Dn > Pn/Do ^b^ | Do < 1 | P_ep_’ < P_UWL_ |
| UWL permeability limited | PL-U | Dn > Pn/Do ^b^ | Do < 1 | P_ep_’ > P_UWL_ |
| Solubility - epithelial membrane permeability limited | SL-E | Dn > Pn/Do | Do > 1 | P_ep_’ < P_UWL_ |
| Solubility - UWL permeability limited | SL-U | Dn > Pn/Do | Do > 1 | P_ep_’ > P_UWL_ |

^a.^ Formerly referred to as the Fa classification system (FaCS); ^b.^ When Do < 1, Do is set as 1.

**Reference**

1. Sugano, K., Calculation of fraction of dose absorbed: comparison between analytical solution based on one compartment steady state concentration approximation and dynamic seven compartment model. *CBI J.* **2009**, *9*, 75–93.

2. Oh, D.M.; Curl, R.L.; Amidon, G.L., Estimating the fraction dose absorbed from suspensions of poorly soluble compounds in humans: a mathematical model. *Pharm. Res.* **1993,** *10*, 264–70.

3. Sugano, K., Introduction to computational oral absorption simulation. *Expert. Opin. Drug Metab. Toxicol.* **2009,** *5*, 259–93.

4. Glomme, A.; März, J.; Dressman, J.B., Predicting the Intestinal Solubility of Poorly Soluble Drugs. In *Testa B, Krämer SD, Wunderli-Allenspach H, Folkers G (Eds.), Pharmacokinetic Profiling in Drug Research, Wiley‐VCH, Weinheim* 2006, pp. 259-280.

5. Sugano, K.; Okazaki, A.; Sugimoto, S.; Tavornvipas, S.; Omura, A.; Mano, T., Solubility and dissolution profile assessment in drug discovery. *Drug Metab Pharmacokinet.* **2007,** *22*, 225–54.

6. Avdeef, A., Leakiness and size exclusion of paracellular channels in cultured epithelial cell monolayers-interlaboratory comparison. *Pharm Res.* **2010,** *27*, 480–9.

7. Takesawa, S.; Ozawa, K.; Mimura, R.; Sakai, K., Mechanism of membrane permeability of solute in the dialyzer. *Jpn J Artif Organs.* **1984**, *13*, 1460–1467.

8. Sugano, K., Estimation of effective intestinal membrane permeability considering bile micelle solubilisation. *Int J Pharm.* **2009**, *368*, 116–122.

9. Sugano, K.; Nabuchi, Y.; Machida, M.; Aso, Y., Prediction of human intestinal permeability using artificial membrane permeability. *Int J Pharm.* **2003,** *257*, 245–51.

10. Sugano, K.; Takata, N.; Machida, M.; Saitoh, K.; Terada, K., Prediction of passive intestinal absorption using bio-mimetic artificial membrane permeation assay and the paracellular pathway model. *Int J Pharm.* **2002,** *241*, 241–51.

11. Sugano, K., Theoretical investigation of passive intestinal membrane permeability using Monte Carlo method to generate drug-like molecule population. *Int J Pharm.* **2009,** *373*, 55–61.

12. Sugano, K., Possible reduction of effective thickness of intestinal unstirred water layer by particle drifting effect. *Int J Pharm.* **2010,** *387*, 103–109.

13. Sugano, K.; Kataoka, M.; Mathews Cda, C.; Yamashita, S., Prediction of food effect by bile micelles on oral drug absorption considering free fraction in intestinal fluid. *Eur J Pharm Sci.* **2010,** *40*, 118–24.

14. Sugano, K.; Terada, K., Rate‐and Extent‐Limiting Factors of Oral Drug Absorption: Theory and Applications. *J Pharm Sci.* **2015**, *104*, 2777–2788.
