# Supplementary material for: Bottom-Up Physiologically Based Oral Absorption Modeling of Free Weak Base Drugs
Source: Pharmaceutics. 2020 Sep 3;12(9):844. doi: 10.3390/pharmaceutics12090844 (PMC7558956; doi:10.3390/pharmaceutics12090844)
Supplement: Supplementary file 1 [file pharmaceutics-12-00844-s001.zip › Supplementary Materials/Supplementary Materials 2_ In vivo Fa values and references.docx]

Supplementary Materials: Bottom-Up Physiologically Based Oral Absorption Modeling of Free Weak Base Drugs

Naoya Matsumura, Asami Ono, Yoshiyuki Akiyama, Takuya Fujita and Kiyohiko Sugano

**Table S2.** Fa data, calculation method, and references.

| **No.** | **Drugs** | **Species/State** | **Dose**  **(mg)** | **D50**  **(μm)** | **Fa** | **AUCr** | **Method ^d^** | **Ref** |
| --- | --- | --- | --- | --- | --- | --- | --- | --- |
| 1 | Acalabrutinib | Human, Fasted | 100 | 98 |  |  |  | [1] |
| 2 |  | Human, Fasted, ARA | 100 | 98 |  | 0.56 |  |  |
| 3 | Albendazole | Human, Fasted | 350 | 3.4 | 0.11 |  | II | [2–4] |
| 4 |  | Human, Fasted | 400 | 3.4 | 0.06 |  | II |  |
| 5 |  | Human, Fed | 400 | 3.4 | 0.29 |  | II |  |
| 6 |  | Human, Fasted | 700 | 3.4 | 0.06 |  | II |  |
| 7 |  | Human, Fasted | 800 | 3.4 | 0.10 |  | II |  |
| 8 |  | Human, Fasted | 1400 | 3.4 | 0.04 |  | II |  |
| 9 |  | Human, Fasted, ARA | 1400 | 3.4 | 0.03 | 0.72 | II |  |
| 10 |  | Human, Fasted | 2100 | 3.4 | 0.03 |  | II |  |
| 11 | Aprepitant | Human, Fasted | 40 | 0.12 | 0.90 |  | III, VI | [5–7] |
| 12 |  | Human, Fasted | 100 | 5.00 | 0.22 |  | III, VI |  |
| 13 |  | Human, Fasted | 125 | 0.12 | 0.79 |  | III, VI |  |
| 14 |  | Human, Fasted | 250 | 0.12 | 0.86 |  | III, VI |  |
| 15 |  | Human, Fasted | 375 | 0.12 | 0.67 |  | III, VI |  |
| 16 |  | Human, Fasted | 500 | 0.12 | 0.83 |  | III, VI |  |
| 17 |  | Human, Fed | 125 | 0.12 | 1.00 |  | III, VI |  |
| 18 |  | Dog, Fasted | 20 | 0.12 | 1.00 |  | III^a^ |  |
| 19 |  | Dog, Fed | 20 | 0.12 | 1.00 |  | III^a^ |  |
| 20 |  | Dog, Fasted | 20 | 0.48 | 0.43 |  | II |  |
| 21 |  | Dog, Fasted | 20 | 1.85 | 0.31 |  | III^a^ |  |
| 22 |  | Dog, Fasted | 20 | 2.0 | 0.33 |  | II |  |
| 23 |  | Dog, Fasted | 20 | 5.0 | 0.28 |  | II |  |
| 24 |  | Dog, Fasted | 20 | 5.5 | 0.24 |  | III^a^ |  |
| 25 |  | Dog, Fed | 20 | 5.5 | 0.77 |  | III^a^ |  |
| 26 |  | Dog, Fasted | 20 | 25.0 | 0.18 |  | II |  |
| 27 | BMS | Human, Fasted | 150 | 3 |  |  |  | [8] |
| 28 |  | Human, Fasted, ARA | 150 | 3 |  | 0.43 |  |  |
| 29 |  | Dog, Fasted | 150 | 40 |  |  |  |  |
| 30 |  | Dog, Fasted, ARA | 150 | 40 |  | 0.03 |  |  |
| 31 | Cinnarizine | Human, Fasted | 25 | 25 | 0.59 |  | III | [9–12] |
| 32 |  | Human, Fasted, ARA | 25 | 25 | 0.16 | 0.27 | III |  |
| 33 |  | Human, Fasted | 25 | 60 | 0.55 |  | III |  |
| 34 |  | Human, Fasted, ARA | 25 | 60 | 0.08 | 0.14 | III |  |
| 35 |  | Human, Fasted | 50 | 25 | 0.81 |  | III |  |
| 36 |  | Human, Fed | 50 | 25 | 1.00 |  | III |  |
| 37 |  | Human, Fasted | 50 | 60 | 0.55 |  | III |  |
| 38 |  | Human, Fed | 50 | 60 | 0.95 |  | III |  |
| 39 |  | Dog, Fasted | 25 | 25 | 0.54 |  | II |  |
| 40 |  | Dog, Fasted, ARA | 25 | 25 | 0.03 | 0.05 | II |  |
| 41 | Danixirin | Human, Fed | 50 | 2 | 0.35 |  | -^b^ | [13,14] |
| 42 |  | Human, Fasted | 100 | 2 | 0.71 |  | -^b^ |  |
| 43 |  | Human, Fasted, ARA | 100 | 2 | 0.30 | 0.43 | -^b^ |  |
| 44 |  | Human, Fed | 100 | 2 | 0.61 |  | -^b^ |  |
| 45 | Dasatinib | Human, Fasted | 50 | 27 | 0.85 |  | II | [15] |
| 46 |  | Human, Fasted | 70 | 27 | 0.98 |  | II |  |
| 47 |  | Human, Fasted | 100 | 27 | 0.90 |  | II |  |
| 48 |  | Human, Fasted, ARA | 100 | 27 | 0.48 | 0.54 | II |  |
| 49 | Dipyridamole | Human, Fasted | 50 | 75 ^c^ | 0.50 |  | VI | [16,17] |
| 50 |  | Human, Fasted, ARA | 50 | 75 ^c^ | 0.31 | 0.63 | VI |  |
| 51 |  | Dog, Fasted | 50 | 75 ^c^ |  |  |  |  |
| 52 |  | Dog, Fasted, ARA | 50 | 75 ^c^ |  | 0.11 |  |  |
| 53 | Enoxacin | Human, Fasted | 400 | 25 ^c^ | 1.00 |  | VI | [18–23] |
| 54 |  | Human, Fed | 400 | 25 ^c^ | 1.00 |  | VI |  |
| 55 |  | Human, Fasted, ARA | 400 | 25 ^c^ | 0.67 | 0.67 | VI |  |
| 56 | Etoricoxib | Human, Fasted | 120 | 40 | 1.00 |  | VI | [24–26] |
| 57 |  | Human, Fasted, ARA | 120 | 40 | 1.00 | 1 | VI |  |
| 58 | Gefitinib | Human, Fasted | 50 | 30 ^c^ | 0.74 |  | VI | [27,28] |
| 59 |  | Human, Fasted | 100 | 30 ^c^ | 0.69 |  | VI |  |
| 60 |  | Human, Fasted | 250 | 30 ^c^ | 0.78 |  | VI |  |
| 61 |  | Human, Fasted, ARA | 250 | 30 ^c^ | 0.41 | 0.53 | VI |  |
| 62 |  | Human, Fed | 250 | 30 ^c^ | 0.80 |  | VI |  |
| 63 |  | Human, Fasted | 500 | 30 ^c^ | 0.80 |  | VI |  |
| 64 | Iburtinib | Human, Fasted | 560 | 10 | 0.21 |  | VI | [29,30] |
| 65 |  | Human, Fasted, ARA | 560 | 10 | 0.18 | 0.87 | VI |  |
| 66 |  | Human, Fed | 560 | 10 | 0.30 |  | VI |  |
| 67 | Ketoconazole | Human, Fasted | 200 | 25 | 0.72 |  | II, III | [17,31–35] |
| 68 |  | Human, Fasted, ARA | 200 | 25 | 0.06 | 0.09 | II, III |  |
| 69 |  | Human, Fed | 200 | 25 | 0.56 |  | II, III |  |
| 70 |  | Human, Fasted | 400 | 25 | 0.92 |  | II, III |  |
| 71 |  | Human, Fed | 400 | 25 | 1.00 |  | II, III |  |
| 72 |  | Human, Fasted | 800 | 25 | 0.86 |  | II, III |  |
| 73 |  | Human, Fed | 800 | 25 | 0.87 |  | II, III |  |
| 74 |  | Dog, Fasted | 200 | 25 |  |  |  |  |
| 75 |  | Dog, Fasted, ARA | 200 | 25 |  | 0.03 |  |  |
| 76 | Palbociclib | Human, Fasted | 125 | 16 | 0.90 |  | II, III, VI | [36] |
| 77 |  | Human, Fasted, ARA | 125 | 16 | 0.47 | 0.52 | II, III, VI |  |
| 78 |  | Human, Fed | 125 | 16 | 0.95 |  | II, III, VI |  |
| 79 |  | Human, Fed, ARA | 125 | 16 | 0.86 | 0.91 | II, III, VI |  |
| 80 | Posaconazole | Human, Fed | 50 | 1.7 | 0.57 |  | VI | [37–42] |
| 81 |  | Human, Fed | 100 | 1.7 | 0.75 |  | VI |  |
| 82 |  | Human, Fasted | 200 | 1.7 | 0.19 |  | VI |  |
| 83 |  | Human, Fasted, ARA | 200 | 1.7 | 0.18 | 0.93 | VI |  |
| 84 |  | Human, Fed | 200 | 1.7 | 0.58 |  | VI |  |
| 85 |  | Human, Fed, ARA | 200 | 1.7 | 0.49 | 0.85 | VI |  |
| 86 |  | Human, Fasted | 400 | 1.7 | 0.14 |  | VI |  |
| 87 |  | Human, Fasted, ARA | 400 | 1.7 | 0.09 | 0.66 | VI |  |
| 88 |  | Human, Fed | 400 | 1.7 | 0.52 |  | VI |  |
| 89 |  | Human, Fed | 800 | 1.7 | 0.42 |  | VI |  |
| 90 |  | Human, Fed | 1200 | 1.7 | 0.23 |  | VI |  |
| 91 |  | Dog, Fasted | 100 | 1.7 | 0.11 |  | VI |  |
| 92 |  | Dog, Fed | 100 | 1.7 | 0.40 |  | VI |  |
| 93 |  | Dog, Fed | 400 | 1.7 | 0.40 |  | VI |  |
| 94 |  | Dog, Fed | 800 | 1.7 | 0.32 |  | VI |  |
| 95 |  | Dog, Fed | 1200 | 1.7 | 0.24 |  | VI |  |

^a^ vs. Nanocrystal formulation; ^b^ vs. Salt; ^c^ Back estimated from the dissolution data; ^d^ See the main document

References

1. Pepin, X.J.H.; Moir, A.J.; Mann, J.C.; Sanderson, N.J.; Barker, R.; Meehan, E.; Plumb, A.P.; Bailey, G.R.; Murphy, D.S.; Krejsa, C.M.; et al. Bridging in vitro dissolution and in vivo exposure for acalabrutinib. Part II. A mechanistic PBPK model for IR formulation comparison, proton pump inhibitor drug interactions, and administration with acidic juices. *Eur. J. Pharm. Biopharm.* **2019**, *142*, 435–448.

2. Schipper, H.G.; Koopmans, R.P.; Nagy, J.; Butter, J.J.; Kager, P.A.; Van Boxtel, C.J. Effect of dose increase or cimetidine co-administration on albendazole bioavailability. *Am. J. Trop. Med. Hyg.* **2000**, *63*, 270–273.

3. Relative Bioavailability of Three Newly Developed Albendazole Formulations: A Randomized Crossover Study with Healthy Volunteers. *Antimicrob. Agents Chemother.* **2004**, *48*, 1051–1054, doi:10.1128/AAC.48.3.1051-1054.2004.

4. Increased systemic availability of albendazole when taken with a fatty meal. *Eur. J. Clin. Pharmacol.* **1988**, *34*, 315–317, doi:10.1007/BF00540964.

5. Wu, Y.; Loper, A.; Landis, E.; Hettrick, L.; Novak, L.; Lynn, K.; Chen, C.; Thompson, K.; Higgins, R.; Batra, U.; et al. The role of biopharmaceutics in the development of a clinical nanoparticle formulation of MK-0869: A Beagle dog model predicts improved bioavailability and diminished food effect on absorption in human. *Int. J. Pharm.* **2004**, *285*, 135–146, doi:10.1016/j.ijpharm.2004.08.001.

6. Takano, R.; Furumoto, K.; Shiraki, K.; Takata, N.; Hayashi, Y.; Aso, Y.; Yamashita, S. Rate-limiting steps of oral absorption for poorly water-soluble drugs in dogs; prediction from a miniscale dissolution test and a physiologically-based computer simulation. *Pharm. Res.* **2008**, *25*, 2334–2344, doi:10.1007/s11095-008-9637-9.

7. Aprepitant PMDA approval document Available online: https://www.pmda.go.jp/drugs/2012/P201200088/index.html (accessed on 10 July 2020).

8. Gesenberg, C.; Mathias, N.R.; Xu, Y.; Crison, J.; Savant, I.; Saari, A.; Good, D.J.; Hemenway, J.N.; Narang, A.S.; Schartman, R.R.; et al. Utilization of In Vitro, In Vivo and In Silico Tools to Evaluate the pH-Dependent Absorption of a BCS Class II Compound and Identify a pH-Effect Mitigating Strategy. *Pharm. Res.* **2019**, *36*, 164.

9. Yamada, I.; Goda, T.; Kawata, M.; Ogawa, K. Application of gastric acidity-controlled beagle dog to bioavailability study of cinnarizine. *Yakugaku Zasshi J. Pharm. Soc. Japan* **1990**, *110*, 280–285.

10. Changxiao, W.G.X.S.L. Effect of food on bioavailability of cinnarizine capsules. *Chinese J. Clin. Pharmacol. Ther.* **1997**, *4*.

11. Ogata, H.; Aoyagi, N.; Kaniwa, N.; Ejima, A.; Sekine, N.; Kitamura, M.; Inoue, Y. Gastric acidity dependent bioavailability of cinnarizine from two commercial capsules in healthy volunteers. *Int. J. Pharm.* **1986**, *29*, 113–120, doi:10.1016/0378-5173(86)90108-0.

12. TOKUMURA, T.; TsusHiMA, Y.; TATSUISHI, K.; KAYANO, M.; MACHIDA, Y.; NAGAI, T. Evaluation of bioavailability upon oral administration of cinnarizine-$β$-cyclodextrin inclusion complex to beagle dogs. *Chem. Pharm. Bull.* **1985**, *33*, 2962–2967.

13. Miller, B.E.; Mistry, S.; Smart, K.; Connolly, P.; Carpenter, D.C.; Cooray, H.; Bloomer, J.C.; Tal-Singer, R.; Lazaar, A.L. The pharmacokinetics and pharmacodynamics of danirixin (GSK1325756) - a selective CXCR2 antagonist - in healthy adult subjects. *BMC Pharmacol. Toxicol.* **2015**, *16*, 1–12, doi:10.1186/s40360-015-0017-x.

14. Bloomer, J.C.; Ambery, C.; Miller, B.E.; Connolly, P.; Garden, H.; Henley, N.; Hodnett, N.; Keel, S.; Kreindler, J.L.; Lloyd, R.S.; et al. Identification and characterisation of a salt form of Danirixin with reduced pharmacokinetic variability in patient populations. *Eur. J. Pharm. Biopharm.* **2017**, *117*, 224–231.

15. Dasatinib drug information Available online: https://www.info.pmda.go.jp/go/pack/4291020F1027_1_15/ (accessed on Jul 10, 2020).

16. Russell, T.L.; Berardi, R.R.; Barnett, J.L.; O’Sullivan, T.L.; Wagner, J.G.; Dressman, J.B. pH-Related Changes in the Absorption of Dipyridamole in the Elderly. *Pharm. Res. An Off. J. Am. Assoc. Pharm. Sci.* 1994, *11*, 136–143.

17. Zhou, R.; Moench, P.; Heran, C.; Lu, X.; Mathias, N.; Faria, T.N.; Wall, D.A.; Hussain, M.A.; Smith, R.L.; Sun, D. pH-Dependent dissolution in Vitro and absorption in Vivo of weakly basic drugs: Development of a canine model. *Pharm. Res.* **2005**, *22*, 188–192, doi:10.1007/s11095-004-1185-3.

18. Chang, T.; Black, A.; Dunky, A.; Wolf, R.; Sedman, A.; Latts, J.; Welling, P.G. Pharmacokinetics of intravenous and oral enoxacin in healthy volunteers. *J. Antimicrob. Chemother.* **2012**, *21*, 49–56, doi:10.1093/jac/21.suppl_b.49.

19. Inhibition of enoxacin absorption by antacids or ranitidine. *Antimicrob. Agents Chemother.* **1989**, *33*, 615–617, doi:10.1128/AAC.33.5.615.

20. Lehto, P.; Kivisto, K. Effects of milk and food on the absorption of enoxacin. *Br. J. Clin. Pharmacol.* **1995**, *39*, 194–196, doi:10.1111/j.1365-2125.1995.tb04431.x.

21. Somogyi, A.A.; Bochner, F.; Keal, J.A.; Rolan, P.E.; Smith, M. Effect of food on enoxacin absorption. *Antimicrob. Agents Chemother.* **1987**, *31*, 638–639.

22. Sekar, V.J.; Lefebvre, E.; De Paepe, E.; De Marez, T.; De Pauw, M.; Parys, W.; Hoetelmans, R.M.W. Pharmacokinetic interaction between darunavir boosted with ritonavir and omeprazole or ranitidine in human immunodeficiency virus-negative healthy volunteers. *Antimicrob. Agents Chemother.* **2007**, *51*, 958–961, doi:10.1128/AAC.01203-06.

23. Lebsack, M.E.; Nix, D.; Ryerson, B.; Toothaker, R.D.; Welage, L.; Norman, A.M.; Schentag, J.J.; Sedman, A.J. Effect of gastric acidity on enoxacin absorption. *Clin. Pharmacol. Ther.* **1992**, *52*, 252–256.

24. Agrawal, N.G.B.; Porras, A.G.; Matthews, C.Z.; Rose, M.J.; Woolf, E.J.; Musser, B.J.; Dynder, A.L.; Mazina, K.E.; Lasseter, K.C.; Hunt, T.L.; et al. Single- and multiple-dose pharmacokinetics of etoricoxib, a selective inhibitor of cyclooxygenase-2, in man. *J. Clin. Pharmacol.* **2003**, *43*, 268–276, doi:10.1177/0091270003251122.

25. Schwartz, J.I.; Agrawal, N.G.B.; Kher, U.A.; DeSmet, M.; Cavanaugh, P.F.; Guillaume, M.; Ebel, D.L.; Merschman, S.A.; Wagner, J.A. Lack of effect of antacids on single-dose pharmacokinetics of etoricoxib. *J. Clin. Pharmacol.* **2007**, *47*, 1342–1347.

26. Mitra, A.; Kesisoglou, F.; Dogterom, P. Application of absorption modeling to predict bioequivalence outcome of two batches of etoricoxib tablets. *AAPS PharmSciTech* **2015**, *16*, 76–84.

27. Hc, S.; Rp, S.; Laight, A.; Dj, K.; Ranson, M.; Ch, W.; Duvauchelle, T. Swaisland 2005 gefitinib Clin Pharmacokinet 44-11 1165-77 Single-dose clinical pharmacokinetic studies of gefitinib . **2005**, *3*, 4–5.

28. Bergman, E.; Forsell, P.; Persson, E.M.; Knutson, L.; Dickinson, P.; Smith, R.; Swaisland, H.; Farmer, M.R.; Cantarini, M. V.; Lennernäs, H. Pharmacokinetics of gefitinib in humans: The influence of gastrointestinal factors. *Int. J. Pharm.* **2007**, *341*, 134–142, doi:10.1016/j.ijpharm.2007.04.002.

29. de Jong, J.; Haddish-Berhane, N.; Hellemans, P.; Jiao, J.; Sukbuntherng, J.; Ouellet, D. The pH-altering agent omeprazole affects rate but not the extent of ibrutinib exposure. *Cancer Chemother. Pharmacol.* **2018**, *82*, 299–308.

30. Gupta, M.K.; Tambwekar, K.; Nair, B.K.; Gole, D.J.; Bernini, M.; Inghelbrecht, S. Compositions Containing Ibrutinib 2016.

31. Lelawongs, P.; Barone, J.A.; Colaizzi, J.L.; Hsuan, A.T.; Mechlinski, W.; Legendre, R.; Guarnieri, J. Effect of food and gastric acidity on absorption of orally administered ketoconazole. *Clin. Pharm.* **1988**, *7*, 228.

32. Pharmacokinetics and dose proportionality of ketoconazole in normal volunteers. *Antimicrob. Agents Chemother.* **1986**, *30*, 206–210, doi:10.1128/AAC.30.2.206.

33. Daneshmend, T.K.; Warnock, D.W.; Ene, M.D.; Johnson, E.M.; Potten, M.R.; Richardson, M.D.; Williamson, P.J. Influence of food on the pharmacokinetics of ketoconazole. *Antimicrob. Agents Chemother.* **1984**, *25*, 1–3.

34. Mannisto, P.T.; Mantyla, R.; Nykanen, S.; Lamminsivu, U.; Ottoila, P. Impairing effect of food on ketoconazole absorption. *Antimicrob. Agents Chemother.* **1982**, *21*, 730–733, doi:10.1128/AAC.21.5.730.

35. Elder, E.J.; Evans, J.C.; Scherzer, B.D.; Hitt, J.E.; Kupperblatt, G.B.; Saghir, S.A.; Markham, D.A. Preparation, characterization, and scale-up of ketoconazole with enhanced dissolution and bioavailability. *Drug Dev. Ind. Pharm.* **2007**, *33*, 755–765, doi:10.1080/03639040601031882.

36. Palbociclib PMDA approval document Available online: https://www.pmda.go.jp/drugs/2017/P20170830001/ (accessed on Jul 10, 2020).

37. Rachel, C.; Sudhakar, P.; Mark, L.; Josephine, L.; Vijay, B. Pharmacokinetics, Safety, and Tolerability of Oral Posaconazole Administered in Single and Multiple Doses in Healthy Adults. *Antimicrob. Agents Chemother.* **2003**, *47*, 2788–2795, doi:10.1128/AAC.47.9.2788.

38. Courtney, R.; Wexler, D.; Radwanski, E.; Lim, J.; Laughlin, M. Effect of food on the relative bioavailability of two oral formulations of posaconazole in healthy adults. *Br. J. Clin. Pharmacol.* **2004**, *57*, 218–222, doi:10.1046/j.1365-2125.2003.01977.x.

39. Courtney, R.; Radwanski, E.; Lim, J.; Laughlin, M. Pharmacokinetics of Posaconazole Coadministered with Antacid in Fasting or Nonfasting Healthy Men. *Antimicrob. Agents Chemother.* **2004**, *48*, 804–808, doi:10.1128/AAC.48.3.804-808.2004.

40. Krishna, G.; Moton, A.; Lei, M.; Medlock, M.M.; McLeod, J. Pharmacokinetics and absorption of posaconazole oral suspension under various gastric conditions in healthy volunteers. *Antimicrob. Agents Chemother.* **2009**, *53*, 958–966, doi:10.1128/AAC.01034-08.

41. Walravens, J.; Brouwers, J.; Spriet, I.; Tack, J.; Annaert, P.; Augustijns, P. Effect of pH and Comedication on Gastrointestinal Absorption of Posaconazole. *Clin. Pharmacokinet.* **2011**, *50*, 725–734, doi:10.2165/11592630-000000000-00000.

42. A.A., N.; P., K.; M.J., H.; S., G.; D., L.; A., C.; R., H.; G.H., M.; C.-C., L.; M.N., C. Pharmacokinetics of SCH 56592, a new azole broad-spectrum antifungal agent, in mice, rats, rabbits, dogs, and cynomolgus monkeys. *Antimicrob. Agents Chemother.* **2000**, *44*, 727–731.
